# Injectable Thermosensitive Thiol-Modified NIPAAm-*g*-Chitosan Hydrogels for Cartilage Regeneration in a Rabbit Osteoarthritis Model

**DOI:** 10.1021/acsomega.4c10829

**Published:** 2025-02-19

**Authors:** Paula Carmela O. Ching, Ya-Ching Chang, Chen-Hsun Weng, Jun-Sheng Wang, Ming-Long Yeh

**Affiliations:** † Department of Biomedical Engineering, National Cheng Kung University, Tainan 70101, Taiwan; ‡ School of Chemical, Biological, and Materials Engineering and Sciences, Mapua University, Manila 1002, Philippines; § Medical Device Innovation Center, National Cheng Kung University, Tainan 70101, Taiwan; ∥ National Applied Research Laboratories, Taiwan Instrument Research Institute, Tainan 300092, Taiwan

## Abstract

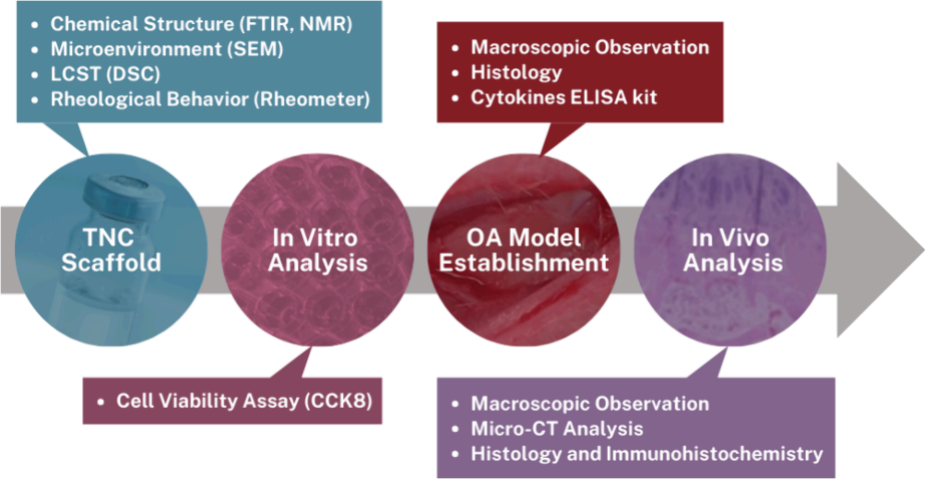

Cartilage tissue has a limited intrinsic capacity for self-healing. Over the decades, researchers have extensively researched methods of cartilage repair, yet some limitations still need to be resolved. Most studies typically evaluate osteochondral regeneration in normal animals. However, traumatic articular cartilage defects may eventually result in osteoarthritis (OA), and the relationship between cartilage defects and OA is not independent. Therefore, in this study, the effect of thiol-modified NIPAAm-*g*-chitosan (TNC) hydrogels containing human adipose-derived mesenchymal stem cells (hADMSCs), with or without etanercept, a TNF-α inhibitor, was evaluated for cartilage regeneration in a monosodium iodoacetate (MIA)-induced OA rabbit model. TNC hydrogels, with a suitable lower critical solution temperature (LCST), porous interior microstructures, enhanced mechanical properties, and without cytotoxicity were synthesized and characterized by DSC, SEM, NMR, and the CCK8 kit. The OA rabbit models were established by MIA injection in the rabbit knees and verified with histological examinations and cytokine detection for IL-1β, IL-6, and TNF-α. According to macroscopic evaluations, micro-CT analysis, and histological and immunohistochemical evaluations, the results of cartilage repair in OA models showed improvement in cartilage regeneration in the cell-seeded hydrogel groups compared with the empty defect groups. Furthermore, etanercept effectively promoted osteochondral defect repair in the first 4 weeks. In OA models, TNC hydrogels containing hADMSCs and etanercepts could be promising for cartilage tissue engineering.

## 1 Introduction

Researchers have extensively studied methods of cartilage repair over decades due to the limited intrinsic capacity of cartilage tissue for self-healing. Traumatic defects and degenerative lesions of articular cartilage, if delayed in treatment compounded with progressive wear-and-tear, may eventually result in osteoarthritis (OA).^1^ Severe trauma and physical diseases, such as OA, commonly result in osteochondral defects.^2^ This disease may lead to cartilage degradation, bone remodeling, and synovium inflammation.^3^

Previous studies have shown that mediating cytokines, including interleukin-1 beta (IL-1β), tumor necrosis factor (TNF) alpha, IL-6, IL-15, IL-17, and IL-18, are upregulated in OA. These cytokines synergistically affect signaling pathways that increase inflammation and cartilage degradation.^4^ In addition, the water content in OA becomes more than 90% due to increased permeability and matrix disruption, which directly lower the elastic modulus. It thus reduces the load-bearing capability of the articular cartilage.^1^

Researchers have developed extensive animal OA models over the past 50 years to gain insights into the onset and progression of OA. Animal OA models can be broadly divided into spontaneous models (naturally occurring or genetically modified) and induced models (surgically or chemically induced).^5^ Animals that develop OA spontaneously, such as mice, rabbits, guinea pigs, and horses, are used as naturally occurring models, which show the best representation of primary OA in humans. However, these are time-consuming and costly.^6,7^ In genetically modified models, the effect of a single gene can be investigated.^8^ However, these may induce additional cartilage abnormalities and oversimplify the disease process because OA is polygenic and costs a lot. Induced models enable rapid studies but do not mimic natural degenerative OA in humans.^9^ In surgically induced models, anterior cruciate ligament (ACL) surgery is the most common method. The advantages are rapid progress and reproducible results, yet this model is considered a more appropriate reflection of post-traumatic OA than spontaneous OA.^10^ Moreover, studying many animals at once requires a significant amount of time. In chemically induced models, monosodium iodoacetate (MIA), papain, collagenase, and quinolone are the common chemicals used to induce OA. This model is easy to induce, repeatable, and suitable for short-term studies. In addition, it avoids infection in animals by eliminating surgery.^5^ In this study, we used the monosodium iodoacetate (MIA)-induced rabbit model, the most commonly used chemically induced model,^11^ which is a metabolic inhibitor that breaks down the cellular aerobic glycolysis pathway and induces cell death through inhibition of glyceraldehyde-3-phosphate dehydrogenase activity in chondrocytes.^12^ Following intra-articular injection of MIA solution, there is a reduction in the number of chondrocytes, and the histological and morphological articular alterations resemble those observed in human osteoarthritis.^9,12^

Tissue engineering, using principles and methods from engineering, material science, biology, and chemistry, has been an emerging research field for over two decades. Key elements required to form biomimetic tissues that restore, maintain, or enhance the functionality of damaged tissues include the cell source (e.g., stem cells and chondrocytes), scaffolds (e.g., biodegradable, natural or synthetic materials, polymers, and nanocomposites), and environmental signals, which involve bioactive factors (e.g., growth factors and cytokines) and physical stimuli (e.g., mechanical and electrical). These three components form the core of tissue engineering approaches.^13^

Adipose-derived mesenchymal stem cells (ADMSCs) have attracted increasing attention in cartilage tissue engineering because of their ability to differentiate into the mesodermal lineage,^14^ minimally invasive acquisition, and excellent chondrogenic potential.^15^ In addition to cells, researchers must carefully consider an ideal scaffold for cartilage regeneration. The scaffold should have adequate mechanical strength, possess suitable degradability, promote cell survival and differentiation, facilitate the diffusion of nutrients and metabolites, and integrate with the surrounding cartilage tissue.^16^ Poly(*N*-isopropylacrylamide) (pNIPAAm) is a thermosensitive hydrogel containing hydrophobic side chains and hydrophilic amide bonds. pNIPAAm’s lower critical solution temperature (LCST) is at about 32 °C,^17^ while the human body temperature is around 37 °C, implying that pNIPAAm chains form a gel-like structure after injection.^18^ According to this reversible sol-to-gel phase transition behavior, pNIPAAm has been used in drug delivery^19^ and biomaterial scaffolds.^20^ However, due to some limitations, such as poor mechanical properties, toxic monomers, and nonbiodegradation, many researchers utilized natural materials to modify the characteristics of pNIPAAm.^21,22^ The polymerization of NIPAAm resulted in biocompatible and biodegradable hydrogels. A study utilizing a NIPAAm-based copolymer confirmed its biocompatibility and biodegradability. After 20 days of degradation, no residual mass remained from the hydrogel.^23^ Another study involving chitosan cross-linked with NIPAAm found that while chitosan alone degraded more quickly, the addition of chitosan made the NIPAAm-copolymer degradable but at a slower rate.^24^ Additionally, Wu et al. successfully synthesized thiol-modified NIPAAm-*g*-chitosan (TNC) hydrogels.^25^ TNC hydrogels proved to be a suitable scaffold for cartilage repair due to their thermo-sensitivity, good biocompatibility, and enhanced mechanical properties by disulfide covalent bond cross-linking.^25,26^

Tumor necrosis factor-alpha (TNF-α) is an essential catabolic factor in inflammation and tissue repair for cartilage.^27,28^ It inhibits the ability of mesenchymal stem cells (MSCs) to differentiate into chondroblasts.^29^ Previous studies have also demonstrated that TNF-α induces the upregulation of MMP-1 and MMP-3 expression in chondrocytes, which may cause osteoarthritis^30^ and lead to the activation of target cells, leading to inflammatory and immune response by the release of several cytokines and apoptotic pathway initiation.^31^ Researchers have applied anti-TNF therapy to various types of severe inflammatory diseases, including rheumatoid arthritis, Crohn’s disease, and degenerative disease of the intervertebral disc. Etanercept is one of the most widely used TNF-α inhibitors. It is a soluble biologic that works by blocking the effects of TNF-α, a proinflammatory cytokine often elevated in various inflammatory conditions, such as arthritis.^32^ Etanercept competitively inhibits the binding of TNF-α to its receptors, thereby rendering TNF biologically inactive.^33,34^ TNF-α interferes with the healing process of osteochondral defects by inducing inflammation and cartilage degradation.^35^ By inhibition of TNF-α, etanercept could potentially enhance healing and prevent further damage. Several studies have researched the impact of anti-TNF-α drugs on cartilage. Some research showed that the administration of etanercept promoted the repair of osteochondral defects and that it could be an effective strategy for cartilage tissue engineering.^36,37^

Therefore, in this study, the researchers aim to synthesize injectable thermosensitive TNC hydrogels and characterize their properties and biocompatibility. In addition, the researchers also wanted to evaluate the effect of TNC hydrogels containing human adipose-derived mesenchymal stem cells (hADMSCs), with or without etanercept, on cartilage regeneration in an MIA-induced OA rabbit model.

## 2 Materials and Methods

### 2.1 Materials

Twenty-four 4-month-old New Zealand white male rabbits weighing 2.8–3.2 kg were obtained from the Livestock Research Institute, Tainan, Taiwan. Human adipose-derived mesenchymal stem cells (hADMSCs) were purchased from Taiwan Advance Bio-Pharmaceutical Inc. *N*-isopropylacrylamide (NIPAAm; Sigma-Aldrich, 97% purity, USA), chitosan (Sigma-Aldrich, 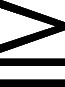
75% deacetylated, 190–375 kDa, USA), ammonium persulfate (APS; Bio-Rad, USA), *N*,*N*,*N*′,*N*′-tetramethylethylenediamine (TEMED; PanReac AppliChem, Germany), *N*-acetyl-l-cysteine (NAC; Sigma-Aldrich, > 99% purity, USA), *N*-hydroxysuccinimide (NHS; Sigma-Aldrich, USA), monosodium iodoacetate (MIA; Sigma-Aldrich, USA), etanercept (Ten-Pack GmbH, Germany), and other commercially available reagents were used as received with no further purification.

The main procedures in this research are TNC synthesis, in vitro analysis of TNC with hADMSCs, and OA model establishment using MIA. Lastly, in vivo analysis using these components, with or without etanercept, was performed to assess articular cartilage repair and regeneration.

### 2.2 Synthesis

#### 2.2.1 Synthesis of NIPAAm-*g*-Chitosan Hydrogels (NC)

The following procedures were performed as previously described.^25^ NIPAAm-*g*-chitosan hydrogels (NC) were synthesized by free radical grafting polymerization.^38^ Briefly, 300 mg of chitosan and 1.5 g of NIPAAm monomers were dissolved in 30 mL of a 1 wt % acetic acid solution for 20 min. The mixture was magnetically stirred and degassed with nitrogen for 30 min. Then, 300 μL of a 0.1% w/v ammonium persulfate (APS) solution and 300 μL of *N*,*N*,*N*′,*N*′-tetramethylethylenediamine (TEMED) were added as the initiator and catalyst. The polymerization mixture was kept at 4 °C for 24 h to ensure chemical reaction completion. The mixture was dialyzed against distilled water by cellulose dialysis membranes (MWCO = 2 kDa) to remove incompletely reacted monomers and chemical reagent residues at 4 °C for 3 days (changing distilled water twice daily). Finally, the dialyzed mixture was lyophilized for 2 days and stored at 4 °C until further use.

#### 2.2.2 Synthesis of Thiol-Modified NIPAAm-*g*-Chitosan Hydrogels (TNC)

The following procedures were performed as previously described.^25^ TNC hydrogels were synthesized by covalent bonds between the carboxyl group of the thiol-containing compound *N*-acetyl-l-cysteine (NAC) and the primary amide groups of chitosan using carbodiimide chemistry.^39,40^ Briefly, 200 mg of lyophilized NIPAAm-*g*-chitosan (NC) cotton-like polymer was dissolved in 10 mL of 1 wt % acetic acid, and 1 M NaOH was used to adjust the pH value to 5. In another container, 800 mg of NAC was dissolved in 4 mL of distilled water, and then 100 mM 1-ethyl-3-(3-(dimethylamino)propyl)carbodiimide (EDC) and 100 mM *N*-hydroxysuccinimide (NHS) were added as cross-linking agents, and the mixture was stirred for 30 min to activate the carboxyl group. The NAC solution was added dropwise into the NIPAAm-*g*-chitosan solution and kept under moderate stirring for 24 h. The mixture was then dialyzed by cellulose dialysis membranes (MWCO 10 kDa) under light protection against several solvents: 5 mM HCl containing 2 μM EDTA overnight, 5 mM HCl containing 2 μM EDTA with 1% sodium chloride for 1 day, 1 mM HCl overnight, and distilled water until pH 7 (changing distilled water twice per day). Finally, the dialyzed mixture was lyophilized for 2 days and stored at 4 °C until further use.

### 2.3 Characterization of NC and TNC Hydrogels

#### 2.3.1 Chemical Structure (FTIR and ^1^H NMR)

Functional groups of pNIPAAm, pure chitosan, lyophilized NC, and lyophilized TNC polymers were identified using Fourier-transform infrared (FTIR) spectroscopy (Jasco, FT/IR-4600, Japan) over the wavenumber range between 4000 and 400 cm^–1^.

The chemical structure of TNC was identified by nuclear magnetic resonance (NMR) ^1^H spectroscopy (Bruker, AVANCE III HD 600 MHz, Germany) using D_2_O as the solvent.

#### 2.3.2 Sol–Gel Temperature

Differential scanning calorimetry (DSC) (PerkinElmer, DSC6000, USA) was employed to observe the lower critical solution temperature (LCST). For sample preparation, lyophilized NC and TNC polymers were hydrated with PBS to 5 wt %. The rising temperature of DSC was set at 2 °C/min from 20 to 50 °C, repeated twice, and the LCST of the samples was defined by the peak temperature from the second cycle.

#### 2.3.3 Microstructure Characterization

The interior microstructures of NC and TNC hydrogels were observed by scanning electron microscopy (SEM; JEOL, JSM-6700F, Japan) under 10 kV and a current of 10 mA. For sample preparation, lyophilized NC and TNC polymers were hydrated with PBS to a 5 wt % polymer solution. Then, 100 μL of the solution was added to a 1.5 mL Eppendorf tube and incubated at 37 °C for 1 day to become gels and achieve swelling equilibrium. Dipping liquid nitrogen immediately into hydrogels caused rapid freezing, and then the frozen hydrogels were lyophilized for 1 day. Afterward, the samples were cut to display the cross-section of the interior structures and coated with platinum (Pt) for 200 s twice for electronic conduction before observation.

#### 2.3.4 Rheological Characterization

The hybrid rheometer (TA Instruments, HR-2, USA) was employed to investigate the rheological properties. In brief, lyophilized NC and TNC polymers were hydrated with PBS to 5 wt %. A 0.5 mL polymer solution was dropped onto the sample plate (40 mm in diameter), and the gap between the parallel sample plates was 200 μm. At 25 and 37 °C, the storage modulus (*G*′) and loss modulus (*G*″) of the samples from 0.1 to 20 Hz were measured by a constant-temperature oscillatory frequency sweep test with 1% strain of the linear viscoelastic region (LVR).

### 2.4 In Vitro Analysis of NC and TNC Hydrogels

#### 2.4.1 Culture of Human Adipose-Derived Mesenchymal Stem Cells

Human adipose-derived mesenchymal stem cells (hADMSCs) were obtained from Taiwan Advance Bio-Pharmaceutical Inc. The cells were cultured in low-glucose Dulbecco’s Modified Eagle Medium (DMEM) with 10% fetal bovine serum (FBS) and 1% antibiotic-antimycotic (AA) at 37 °C in a 5% CO_2_ incubator, and the culture medium was renewed every 2 days. At approximately 80% confluence, the cells were detached using 1x trypsin. Only cells from passage 3 were used in the following experiments involving cells.

#### 2.4.2 Cell Viability

The cell counting kit-8 (CCK8) was used to calculate cell viability. For sample preparation, lyophilized NC and TNC polymers were sterilized by exposure to UV light for 2 days, then soaked in DMEM without FBS for 24 h at 37 °C to form 0.1 g/mL conditioned media, according to ISO 10993-5 and ISO 10993-12. Afterward, the conditioned media were filtered, and 10% FBS was added. A total of 2000 cells per well were seeded in 96-well plates with normal medium and allowed to attach for 24 h. Following cell attachment, the conditioned media were used to replace the normal medium, and then the media were changed every 2 days. The CCK8 working solution (1:10 ratio in cell medium) was used for colorimetric determination of cell count at days 1, 4, and 7. The absorbance at 450 nm was measured using a microplate reader (Molecular Device, Emax Plus, USA).

### 2.5 In Vivo Osteoarthritis Animal Models

#### 2.5.1 Ethics Statement

All animal procedures were approved by the Animal Center of Chimei Medical Center and carried out under the ethical standards of the International Council for Laboratory Animal Science guidelines.

#### 2.5.2 Animal Surgical Procedure

Twenty-four (total 48 knees) 4-month-old New Zealand white male rabbits (2.8–3.2 kg) from the Livestock Research Institute, Tainan, Taiwan, were used in this study. All rabbits were divided into four groups: the sham group, the empty defect group (ED), the TNC + hADMSCs group, and the TNC + hADMSCs + etanercept group, and two rabbits were used for OA model verification. Besides the sham group, all rabbits (a total of 20 rabbits, 40 knees) were treated with MIA to establish chemically induced OA models. First, MIA powder (Sigma-Aldrich, USA) was dissolved in sterile normal saline to prepare an MIA solution with a concentration of 16 mg/mL.^41,42^ After anesthetization with 0.2 mL/kg of body weight with Zoletil 50 (Virbac, France) and tranquilization with 0.5 mL/kg of body weight with Rompun (Bayer, Germany), both knees were shaved and then injected intra-articularly with 250 μL of MIA solution to induce OA. After 28 days, two normal and two MIA-induced rabbits were sacrificed via a CO_2_ insufflator (Stryker Endoscopy, USA). Joint cartilage and joint fluid were collected for OA model verification.

The remaining MIA-induced OA rabbits were anesthetized and tranquilized; then, both knees were shaved and disinfected. The patella was dislocated laterally, and an incision was made to expose the patellofemoral groove of the knee. A round, full-thickness osteochondral defect (3 mm in diameter and 3 mm in depth) was created in the patellofemoral groove in both knees using a portable electric drill (CAN TA, Taiwan).^43^ In the TNC + hADMSCs group and the TNC + hADMSCs + etanercept group, TNC injectable hydrogels with 4 × 10^7^ cell/mL hADMSCs were injected into the defect and then exposed to a heating lamp for gelation. Then, 125 μg/mL etanercept (Ten-Pack GmbH, Germany) was subcutaneously injected in the TNC + hADMSCs + etanercept group.^36^

After the surgical procedure, the patella was relocated, and then the joint capsule was sutured by an absorbable Vicryl 4-0 suture, followed by Nylon 4-0 to suture the skin layer. Then, 0.25 mL of enrofloxacin (Lerocin 10%) was injected for 3 days to avoid infection. Postsurgery, the rabbits were housed singly and allowed free cage activity with neck collars to prevent wound biting. The rabbits were sacrificed after 4 and 12 weeks, and both knees were harvested for examination.

#### 2.5.3 Verification of OA Model (Macroscopic and Histological Examinations)

After 28 days of MIA injection, the joint cartilage from both knees of two normal rabbits and two MIA-induced rabbits was harvested for OA model verification. All samples were immediately fixed with 10% formalin for 5 days and then decalcified with 10% formic acid for 3 weeks. The samples were sent to the NCKU Hospital tissue bank for sectioning and hematoxylin and eosin staining. Sections of 4 μm thickness were stained with Safranin O-fast green and examined under an optical microscope (Olympus BX41, Japan). The results of the histological evaluation were performed using the OARSI (Osteoarthritis Research Society International) scoring system, with 24 as the maximum score, by three researchers to evaluate the severity of osteoarthritis.^44,45^

#### 2.5.4 Verification of OA Model (Proinflammatory Cytokine Detection)

The collected joint fluids from both knees of two normal rabbits and two MIA-induced rabbits were centrifuged at 4000 rpm for 10 min, and then the supernatant was kept at −20 °C. An ELISA Kit (FineTest, China) was used to detect the inflammatory cytokines IL-1β, IL-6, and TNF-α.

#### 2.5.5 Regeneration of Osteochondral Defect

##### 2.5.5.1 Macroscopic Evaluations

After 4 and 12 weeks, the rabbits were sacrificed, and the joint cartilages of both knees were harvested. The joint surface was washed with PBS and photographed immediately. Three researchers evaluated the macroscopic appearance of the regenerated tissue using the Wayne scoring system, which was modified based on the International Cartilage Repair Society (ICRS) Visual Histological Scale.^46^ The maximum score is 12 points.

##### 2.5.5.2 Micro-CT Analysis

After evaluation of the gross appearance, the samples were fixed with 10% formalin. Before decalcification, micro computed tomography (Micro-CT; Bruker, SkyScan1076 & SkyScan1276, USA) was performed to evaluate qualitative and quantitative bone regeneration levels. The scanning parameters were as follows: source voltage = 50 kV, source current = 200 μA, image pixel size = 18 μm, aluminum filter = 0.5 mm, and rotation angle = 180° with a 0.8° rotation step.

The images were analyzed by CT-An software. From the CT data, a cylindrical region of interest (ROI), 3 mm in diameter, within the repaired site, was selected for analysis. The volume and thickness of the regenerated bone were measured as bone volume per tissue volume (BV/TV) and trabecular thickness (Tb. Th).

##### 2.5.5.3 Histological and Immunohistochemical Analysis

After the Micro-CT analysis, all samples were decalcified with 10% formic acid for 3 weeks. Then, the samples were sent to the NCKU Hospital tissue bank division for sectioning and hematoxylin and eosin staining. Sections of 4 μm thickness were stained with Safranin O-fast green for GAG content and examined under an optical microscope.

To detect the collagen type I (fibrocartilage) and collagen type II (hyaline cartilage) contents in the regenerated tissues, the sections were stained for immunohistochemistry (IHC) analysis (Mouse/Rabbit Probe HRP Labeling Kit, BIOTnA, Taiwan) according to standard protocols. The histological scoring system, reported by Wakitani et al.,^47^ with 15 as the maximum score, was used to evaluate cartilage regeneration.

### 2.6 Statistical Analysis

All data were obtained from at least three independent tests and are presented as mean ± standard deviation (SD). Comparison between values of multiple groups was analyzed using one-way ANOVA. The significance level was set at 95% (*p*-value <0.05), and GraphPad Prism 7 software was used for data analysis.

## 3 Results

### 3.1 Synthesis and Modification of NIPAAm-*g*-Chitosan Hydrogels

**Figure 1:**
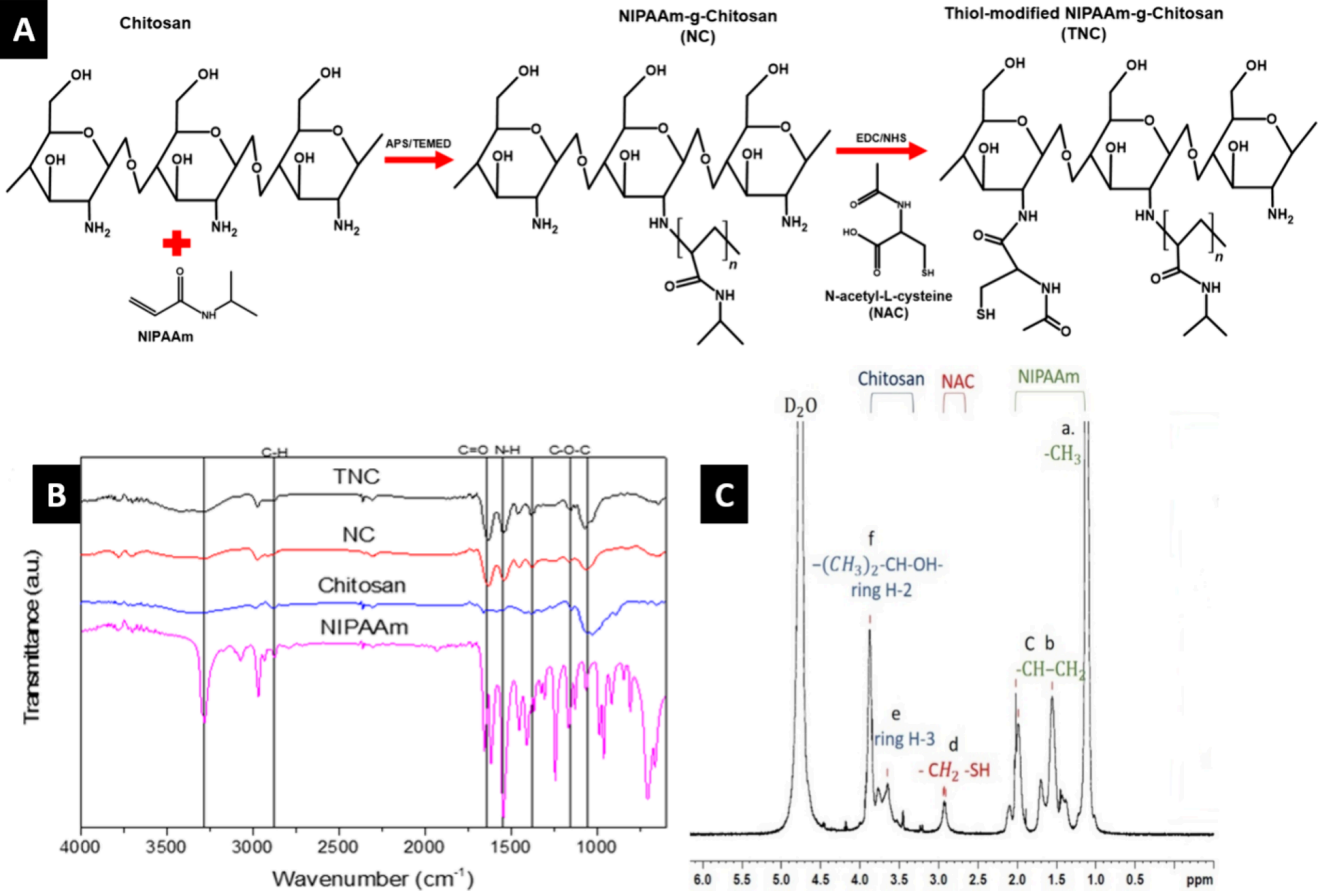
Synthesis and characterization of NC and TNC Hydrogels. (A) Free radical polymerization by chitosan grafting with NIPAAm monomers and the covalent attachment of the NAC to chitosan using carbodiimide. (B) FTIR spectra of NIPAAm, chitosan, NC, and TNC over the wavenumber range between 4000 and 400 cm^–1^. (C) ^1^H NMR spectrum of TNC in the D_2_O solvent. The spectrum showed chemical structures of NIPAAm (a–c), NAC (d), and chitosan (e, f).

For NIPAAm-*g*-chitosan hydrogel (NC) synthesis (Figure 1A), APS was used as an initiator to trigger the free radical polymerization by radical chitosan grafting with NIPAAm monomers, and TEMED was used as a catalyst to increase the reaction rate in the process. Afterward, TNC hydrogels were synthesized by the covalent attachment of the activated carboxyl bonds of the thiol-containing compound NAC to the primary amine groups of chitosan using carbodiimide chemistry from EDC and NHS.

For NC and TNC hydrogel preparation, lyophilized NC and TNC polymers were hydrated with PBS to 5 wt % polymer solutions, showing sol phase at room temperature and gel phase at 37 °C, close to the temperature of the mammals.

### 3.2 Characterization of NC and TNC Hydrogels

#### 3.2.1 Chemical Structure Analysis

The functional groups of various samples were identified using FTIR over the wavenumber range between 4000 and 400 cm^–1^ (Figure 1B). The spectrum revealed transmittance peaks of chitosan including 2895 cm^–1^ (C–H stretch), 1640 cm^–1^ (C=O stretch, amide group), 1560 cm^–1^ (N–H deformation, amino group), 1380 cm^–1^ (C–O stretch, amide group), 1155 cm^–1^ (C–O–C stretch), and 1070 cm^–1^ (C–O stretch) as previously described.^48^ For the NIPAAm group, typical peaks were shown at 3285 cm^–1^ (N–H stretch), 1640 cm^–1^ (C=O stretch, amide group), and 1560 cm^–1^ (N–H deformation, amino group).^49^ The spectra also showed that both typical peaks of chitosan and NIPAAm were exhibited in the NC and TNC groups, such as C–H stretch, C=O stretch, N–H deformation, and C–O–C stretch, which meant the synthesis of NC hydrogels was successful.

To confirm the covalent attachment of NAC, the S–H bond at 2553 cm^–1^ should be observed; however, it was not clearly detected in the IR spectrum due to its weak intensity. Consequently, ^1^H NMR spectroscopy was performed to more accurately identify the thiol groups in the TNC hydrogels (Figure 1C). The ^1^H NMR spectrum of TNC in D_2_O solvent showed methyl peaks (−CH_3_) at 1.12 ppm (a), two broad peaks (−CH–CH_2_−) at 1.56 ppm (b) and 1.99 ppm (c) of NIPAAm.^50^ For chitosan, there was a ring H-3 peak at 3.65 ppm (e), an overlap of isopropyl (−(CH_3_)_2_–CH–OH−), and a ring H-2 peak at 3.87 ppm (f). In chitosan with NAC covalently grafted to the amines, a new resonance peak (−CH_2_–SH) appeared at 2.94 ppm (d), indicating the success of the thiol modification.^39^

#### 3.2.2 Lower Critical Solution Temperature (LCST)

**Figure 2:**
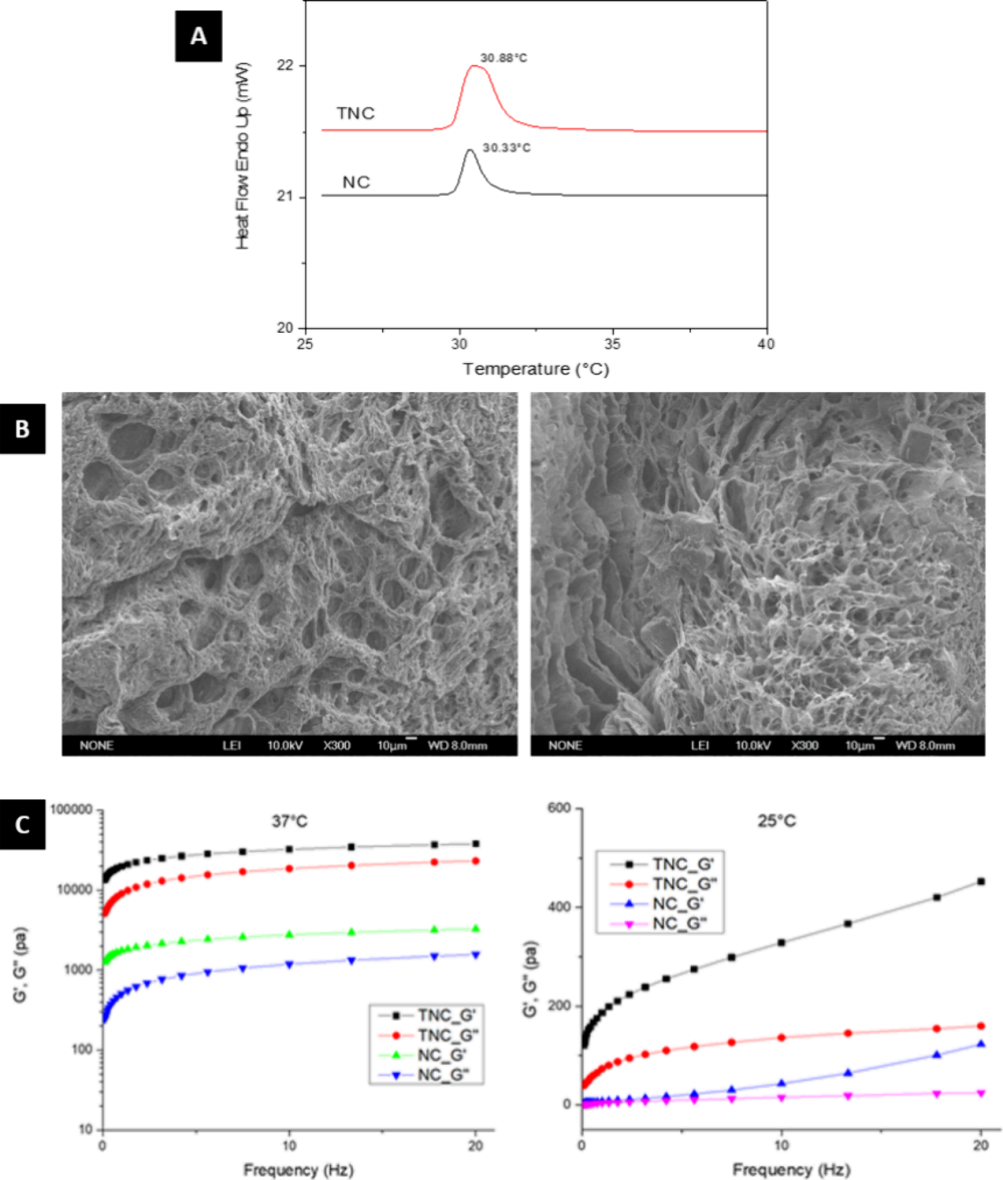
Characterization of NC and TNC hydrogels. (A) DSC thermograms showing the LCST of NC and TNC hydrogels. (B) SEM images of the interior microstructures of NC and TNC hydrogels. The pore size ranged from 10 to 40 μm. Scale bar 10 μm. (C) Storage modulus (*G*′) and loss modulus (*G*″) of NC and TNC hydrogels measured by a constant-temperature oscillatory frequency sweep test from 0.1 to 20 Hz with 1% strain at 37 and 25 °C.

DSC was employed to observe the sol–gel phase transition temperature, known as the lower critical solution temperature (LCST), and the LCST was defined by the peak temperature from the second heating cycle. As shown in Figure 2A, the LCST of NC and TNC hydrogels was 30.33 and 30.88 °C, respectively. There was no significant difference in LCST between the NC and TNC hydrogels. The area under the curve in the DSC plot of TNC though was higher than NC due to the thiol modification in TNC. Thiol modification resulted in a higher molecular weight and increased thermal stability.^51^ Furthermore, the introduction of the thiol group caused improved swelling behavior, cross-linking, biological interactions, and mechanical stability^52^ which could be potential reasons for the higher AUC.

#### 3.2.3 Microenvironment of Hydrogels

Interior microstructures of NC and TNC hydrogels were observed by SEM. As shown in (Figure 2B), NC and TNC hydrogels were both porous, forming a 3-dimensional interconnected microenvironment, which was reported as a crucial requirement in tissue engineering to promote cell attachment and cellular ingrowth.^53^ In addition, compared to NC hydrogels, thiol-modified hydrogels resulted in better cross-linking density, and the pore size was decreased from 40 to 10 μm.

#### 3.2.4 Rheological Behavior

The hybrid rheometer was employed to investigate the rheological properties (storage modulus (*G*′) and loss modulus (*G*″)). As shown in Figure 2C, the storage moduli (*G*′) of NC and TNC groups were much higher than the loss moduli (*G*″), indicating the gel phase of hydrogels at 37 °C. While sweeping more than 5 Hz, the modulus revealed a plain curve, independent of frequency. In addition, the storage modulus (*G*′) of TNC was about 10 times higher than NC. This fact proved that thiol-modified hydrogels successfully improved the mechanical properties by disulfide covalent bond cross-linking.

On the other hand, in NC and TNC groups, both the storage modulus (*G*′) and loss modulus (*G*″) were significantly dropped down at 25 °C. This indicated that the mechanical properties were significantly different between the gel-like phase at 37 °C and the sol-like phase at 25 °C.

### 3.3 In Vitro Analysis of NC and TNC Hydrogels

#### 3.3.1 Cell Viability

**Figure 3:**
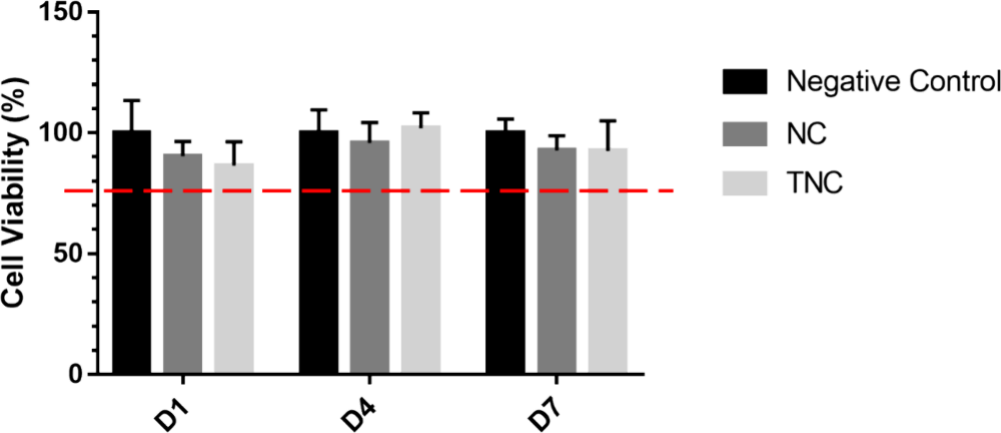
Cell viability of human adipose-derived mesenchymal stem cells in NC and TNC-conditioned medium. There was no significant difference between each group within the same time point.

The cultured passage 3 of hADMSCs was used to test cell viability with the cell counting kit-8 in NC and TNC-conditioned media for 1, 4, and 7 days (Figure 3). The relative cell viability was calculated as the percentage of the culture medium without hydrogels (negative control group). The calculated percentage of cell viability for the NC and TNC groups for all time points was >85%, and there was no significant difference between the negative control and the hydrogel groups within the same time points. Additionally, according to ISO 10993-5, if the relative cell viability of the sample-conditioned medium is 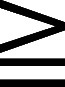 70% of the control group, then the material shall be considered noncytotoxic.

### 3.4 In Vivo Analysis in Animal Model

#### 3.4.1 Verification of OA Model

The knee surfaces of normal rabbits were smooth, shiny, and white compared to the knee of the MIA-induced model in which lesions affect the entire articular surface. The irregular and dull surfaces of the MIA-induced knee were very apparent. However, due to swelling, deformity, and a thickened joint capsule, usually present in OA models, which were not noticeable in our study, we could not observe whether OA was successfully caused by MIA before seeing the surface of the knee. Therefore, we needed to evenly distribute the rabbits so that OA and non-OA rabbits were in each defect group to prevent significant differences between the groups.

**Figure 4:**
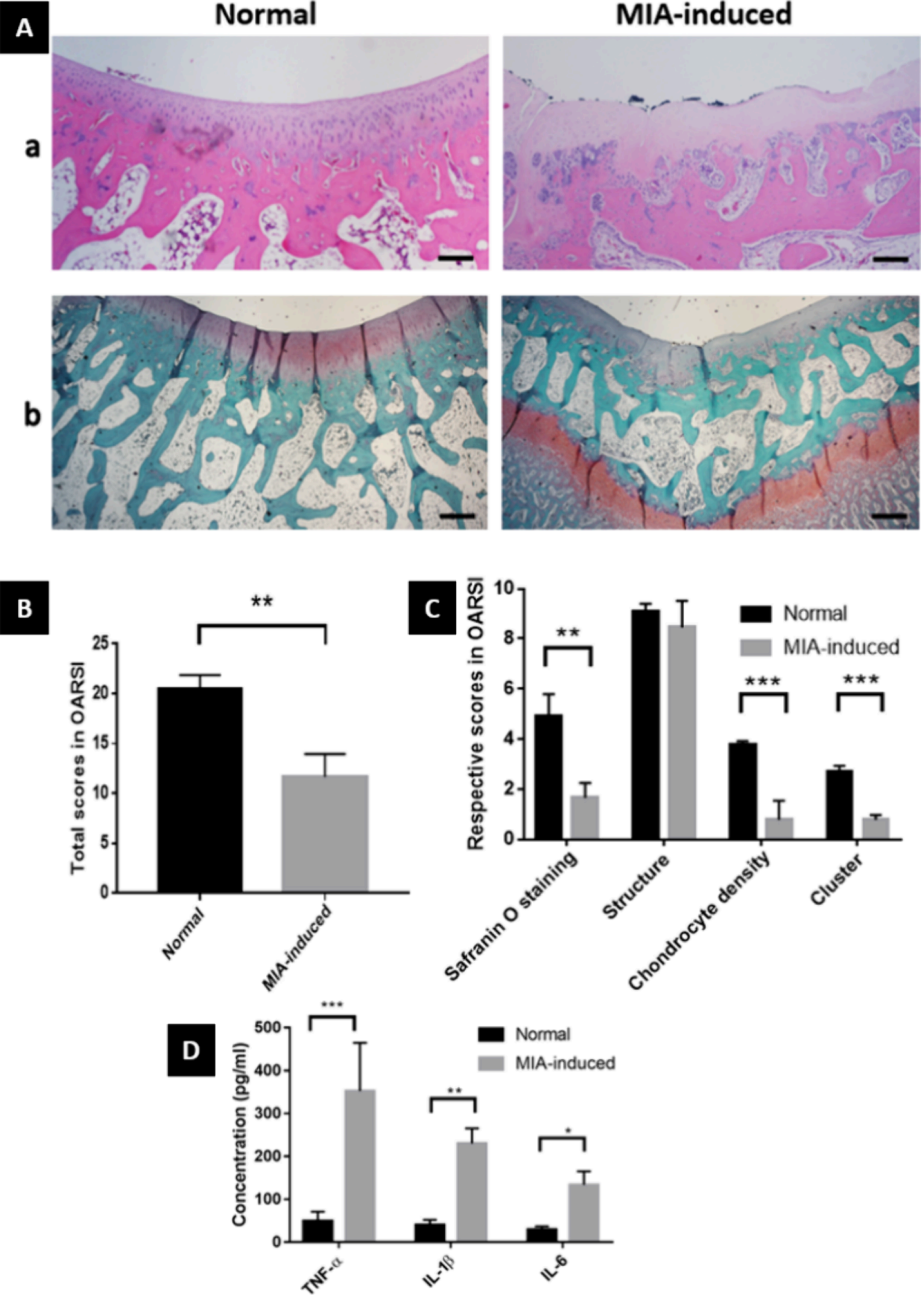
Verification of the induced OA. (A) Histological images with (a) H&E staining and (b) Safranin O-fast green staining. Magnification: 4X; scale bars: 500 μm. (B) Histological evaluation using the OARSI score. (C) Respective results in OARSI score. (D) Expression levels of inflammatory cytokines, TNF-α, IL-1β, and IL-6, in the joint fluid of rabbits. Values reported as means ± SD for *n* = 4 (* *p* < 0.05, ** *p* < 0.005, *** *p* < 0.001).

In H&E staining, the knees of normal rabbits showed no changes (Figure 4A). The surface was intact, and the chondrocytes and chondrocyte columns were arranged regularly. In contrast, irregular surfaces with fissures and erosions, empty lacunae with hypocellularity, and difficulty in observing superficial and middle zones are shown in the MIA-induced rabbit. In Safranin O-fast green staining, chondrocytes were also decreased and disorganized, and the surface was slightly irregular, with minimal cracks in the MIA-induced models. In addition, the coloration of Safranin O-fast green staining was not apparent, indicating decreased glycosaminoglycans (GAGs) in the extracellular matrix. The severity assessment of OA was evaluated using the OARSI score, and the results are shown in Figure 4B,C. There was a significant difference (*p* = 0.0016) between the total scores of normal and MIA-induced knees. However, in the OARSI system, the score of the structure in the OA group was comparable to that of the normal group.

To detect the inflammatory cytokines, TNF-α, IL-1β, and IL-6, ELISA kits were used (Figure 4D). Compared to the levels of TNF-α (48.48 ± 22.72 pg/mL), IL-1β (39.8 ± 12.19 pg/mL), and IL-6 (29.17 ± 7.916 pg/mL) in the joint fluid of the normal group, TNF-α (352.2 ± 112.7 pg/mL), IL-1β (229.9 ± 35.31 pg/mL), and IL-6 (133.6 ± 31.42 pg/mL) in the MIA-induced group were significantly increased, which indicated that the OA models were successfully induced by MIA intra-articular injection.

#### 3.4.2 Regeneration of Osteochondral Defect

**Figure 5:**
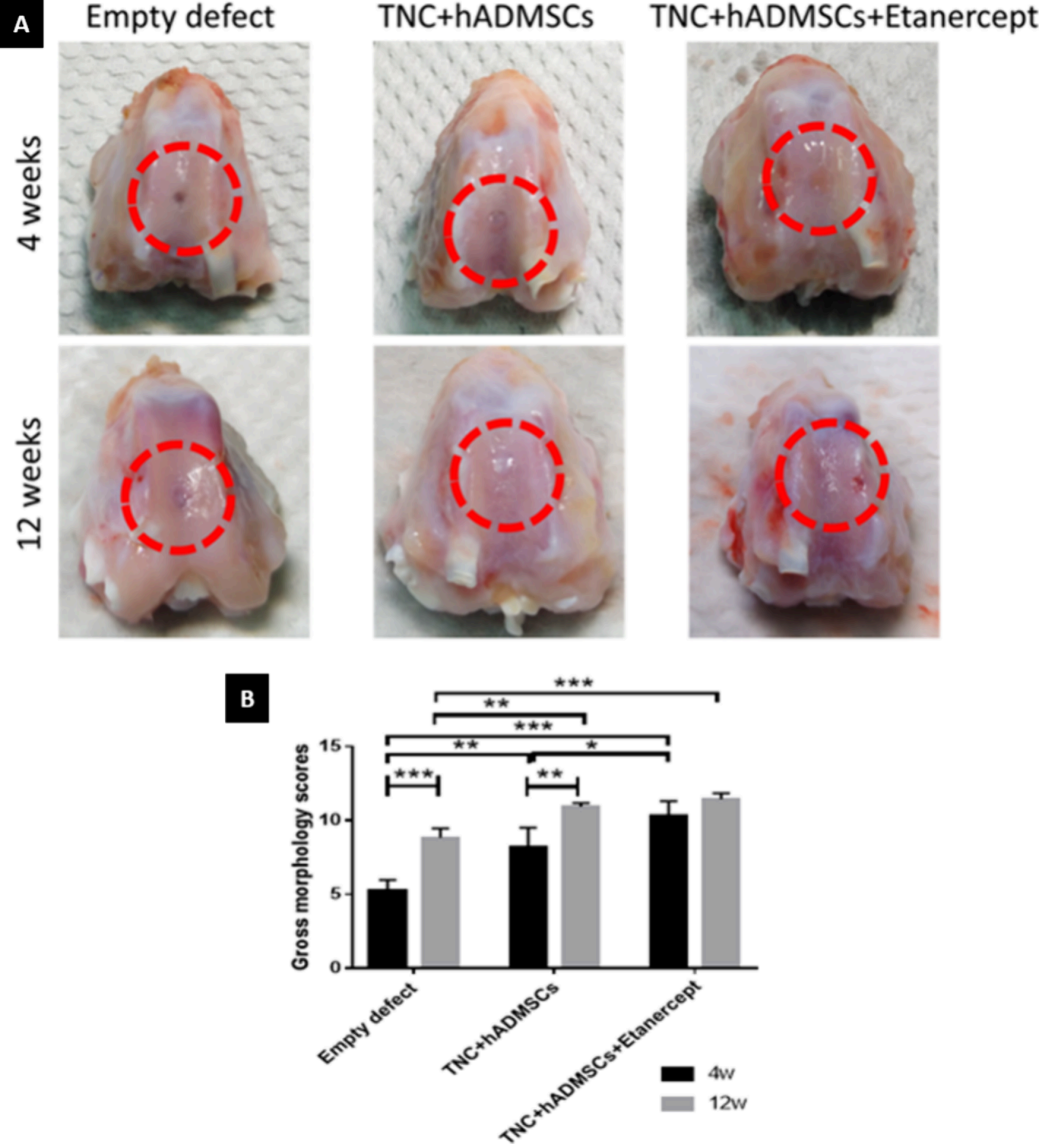
Macroscopic evaluation of tissue regeneration at 4 and 12 weeks after the operation. (A) Gross appearance of the articular cartilage defects at 4 and 12 weeks. (B) Qualitative scores of the gross morphology appearances. Values reported as means ± SD for *n* = 6 (* *p* < 0.05, ** *p* < 0.005, *** *p* < 0.001).

A cartilage defect model was used to evaluate the repair capability of the hydrogels (Figure 5A). In the postoperative 4-week groups, the cartilage defects were still present in the empty defect group, but the repaired tissue with higher coverage can be observed in both cell-seeded hydrogel groups; however, the color of neocartilages was reddish, and the margins of the defects were visible. Moreover, the repaired tissue exhibited a rough surface in all groups. In the TNC + hADMSCs + etanercept group, some knees had better cartilage regeneration under gross observation.

In the postoperative 12-week groups, the empty defect group displayed incomplete tissue coverage compared to the defects in the cell-seeded hydrogel groups, wherein neocartilages, with a similar color to the host tissue, were nearly filled. The neo-tissues in the empty defect group displayed a noticeable margin with the surrounding original cartilage tissue. In contrast, the boundary between the normal tissue and the defect was not apparent in the cell-seeded hydrogel groups. In addition, there were slightly irregular surfaces in all 12-week groups but smoother than those in the 4-week groups.

The macroscopic appearance of the regenerated tissue was assessed using the Wayne scoring system (Figure 5B). The total scores for the 4-week groups were as follows: Empty defects (5.25 ± 0.74), TNC + hADMSCs (8.22 ± 1.30), and TNC + hADMSCs + etanercept (10.28 ± 1.04). A significant improvement in the scores was observed between the empty defect group and the treated group (TNC + hADMSCs). Moreover, the addition of etanercept further increased the total score for the TNC + hADMSCs + etanercept group compared with the other two groups. The scores for the 12-week groups were as follows: Empty defects (8.83 ± 0.64), TNC + hADMSCs (10.94 ± 0.25), and TNC + hADMSCs + etanercept (11.44 ± 0.40). Significant differences were found between the empty defect group and the cell-seeded hydrogel groups. Although no significant differences were observed between the TNC + hADMSCs and TNC + hADMSCs + etanercept groups, both groups scored significantly higher than the empty defect group. Overall, the scores significantly increased from 4 to 12 weeks for all of the groups, except for the etanercept group, which maintained a consistently high score in both the 4- and 12-week evaluations, with little difference between the two time points.

#### 3.4.3 Micro-CT Reconstruction Analysis

**Figure 6:**
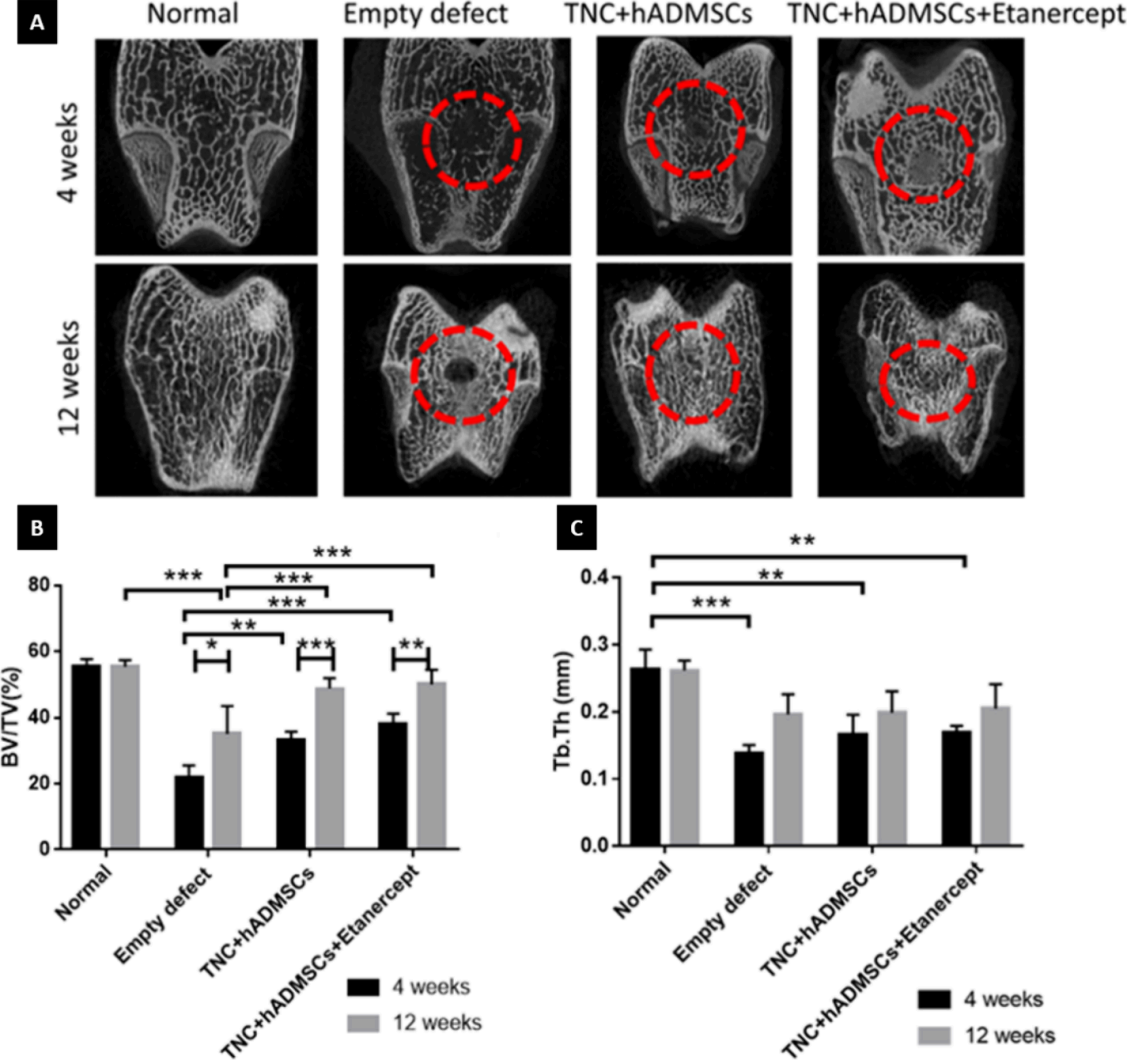
Analysis of bone regeneration at 4 and 12 weeks after the operation. (A) Bone assessment of 2D micro-CT images. (B) Ratio of bone volume to tissue volume (BV/TV). (C) Thickness of trabecular bone (Tb. Th). Values reported as means ± SD for *n* = 6 (* *p* < 0.05, ** *p* < 0.005, *** *p* < 0.001).

Micro-CT was performed to evaluate the qualitative and quantitative bone regeneration levels. In the 2D micro-CT images (Figure 6A), newly synthesized mineral matrices were regenerated in all groups, especially in the cell-seeded hydrogel groups. The areas of bone regeneration were increased in the 12-week groups compared with the 4-week groups. To quantify the bone regeneration levels, the volume and thickness of the regenerated bone were measured as bone volume per tissue volume (BV/TV) and trabecular thickness (Tb. Th) as shown in Figure 6B,C.

The BV/TV (%) values for the 4-week groups were as follows: Normal (55.48 ± 2.20), Empty defect (21.79 ± 3.79), TNC + hADMSCs (33.29 ± 2.51), and TNC + hADMSCs + etanercept (38.17 ± 3.10). Both treated groups exhibited significantly higher bone volume compared to the empty defect group. However, no significant difference was observed between the TNC + hADMSCs and the TNC + hADMSCs + etanercept groups. For the 12-week groups, the BV/TV values were: Normal (55.46 ± 1.95), Empty defect (35.02 ± 8.48), TNC + hADMSCs (48.79 ± 3.16), and TNC + hADMSCs + etanercept (50.05 ± 4.45). Both treated groups showed significantly higher bone volume compared to the empty defect group, but there was no significant difference between the etanercept and nonetanercept groups. Overall, a significant improvement in bone volume was observed between the 4- and 12-week time points for all groups, which corresponded to the gross appearance results. After 12 weeks, although the bone volumes in the TNC + hADMSCs and TNC + hADMSCs + etanercept groups were still lower than that of the normal group, there were no significant differences among the three, indicating that their bone volume was comparable to normal bone density.

The values of Tb. Th (mm) values for the 4-week groups were as follows: Normal (0.263 ± 0.030), Empty defect (0.139 ± 0.012), TNC + hADMSCs (0.166 ± 0.030), and TNC + hADMSCs + etanercept (0.169 ± 0.011). Significant differences were observed between the normal group and all experimental groups. For the 12-week groups, the Tb. Th values were: Normal (0.262 ± 0.015), Empty defect (0.196 ± 0.030), TNC + hADMSCs (0.199 ± 0.032), and TNC + hADMSCs + etanercept (0.205 ± 0.036). No significant difference was found between the normal group and the experimental groups, indicating that the bone thickness in the experimental groups was comparable to that in the normal group by the 12-week time point.

#### 3.4.4 Histological and Immunohistochemical Analysis

**Figure 7:**
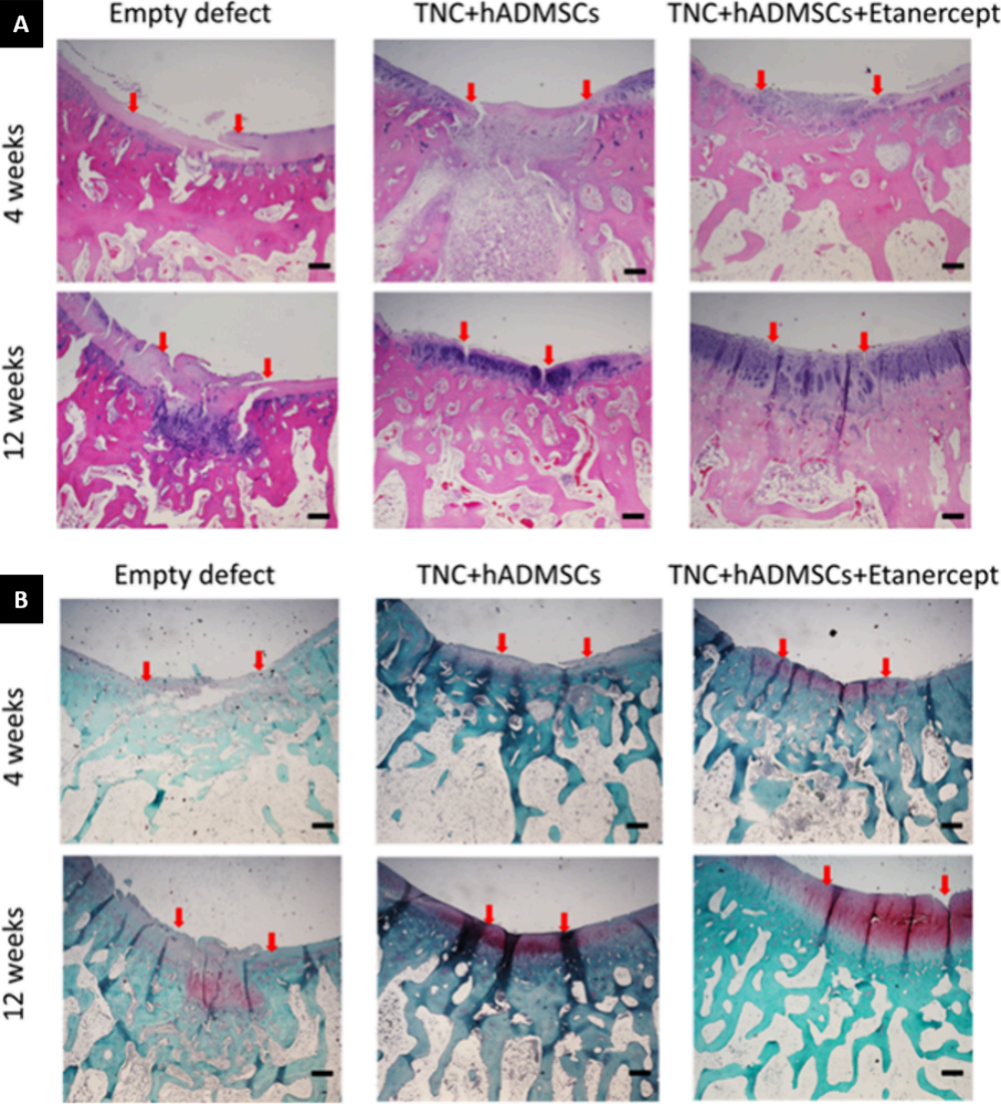
Representative images of histological analysis at 4 and 12 weeks after operation using (A) H&E and (B) Safranin O-fast green staining. Magnification: 4X; scale bars: 500 μm. Red arrows indicate the border of the repaired tissue.

The H&E staining is shown in Figure 7A. In the cell-seeded hydrogel groups of 4 weeks and the empty defect groups, the surfaces were discontinuous, and neo-tissues in the subchondral bone were disorganized. Compared with the 4-week groups, increased cell number, thicker cartilage layers, and higher staining intensity were observed in the 12-week cell-seeded hydrogel groups, especially in the etanercept group. In the TNC + hADMSCs group, after 12 weeks, the reparative tissue in the superficial zone lacked cells, and the boundary with the surrounding native tissue was noticeable. In the TNC + hADMSCs + etanercept group of 12 weeks, the regenerated cartilage was similar to the normal tissue in which the superficial zone appeared intact and contained clusters of chondrocytes arranged in columns in the deep zone. However, the surfaces were irregular in almost all groups.

The Safranin O-fast green staining is shown in Figure 7B. Safranin O-fast green staining was used to stain the proteoglycans. Compared with the 4-week groups, abundant glycosaminoglycan (GAGs) were exhibited in the 12-week groups, especially in the cell-seeded hydrogels groups. The empty defect 12-week group showed GAG formation in the subchondral bone area which corresponded to the site of cells in H&E staining.

**Figure 8:**
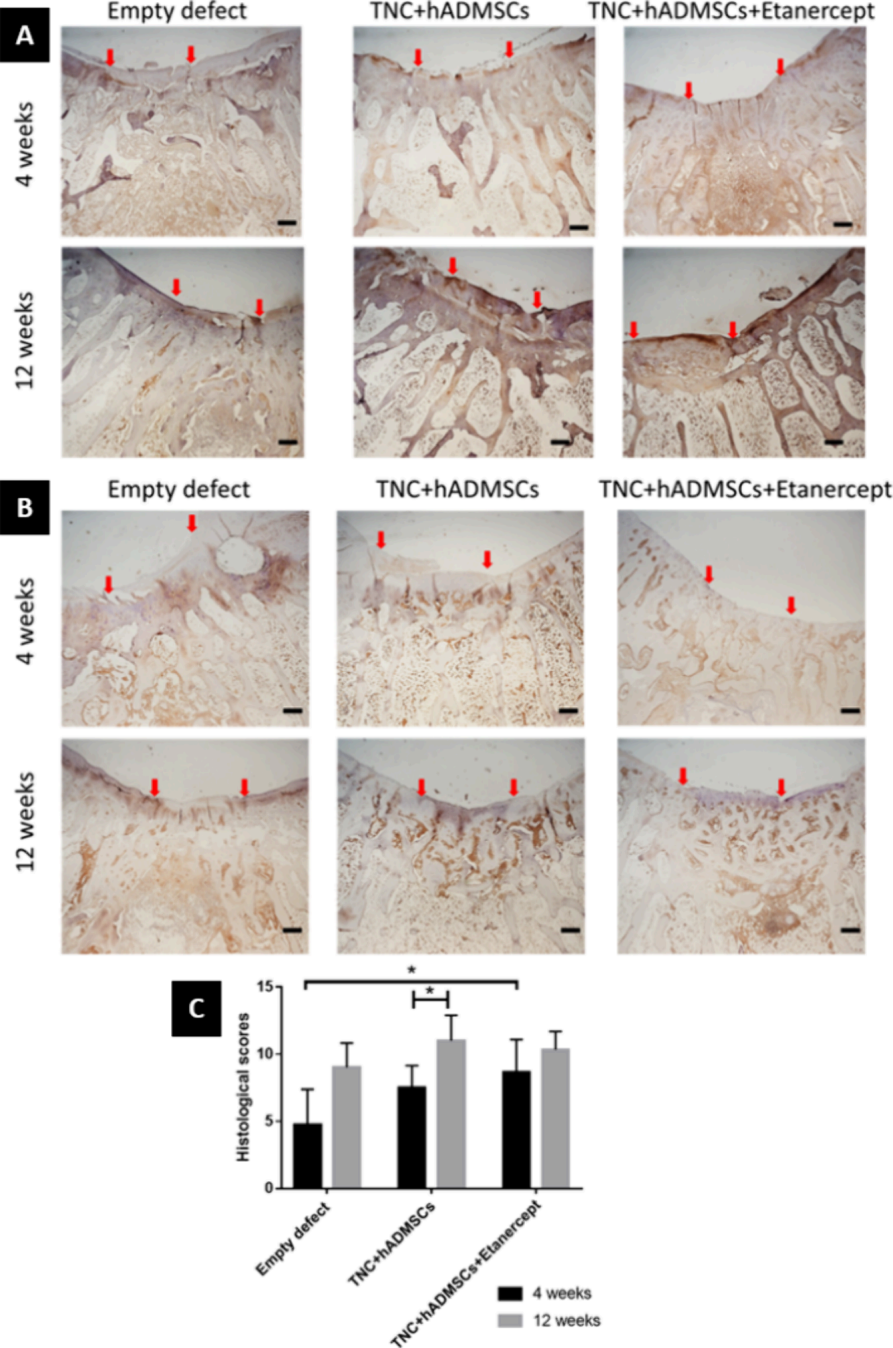
Representative images of immunohistochemistry analysis at 4 and 12 weeks after the operation with (A) anti-Col II antibody and (B) anti-Col I antibody. Magnification: 4X; scale bars: 500 μm. Red arrows indicate the border of the repaired tissue. (C) Histological score chart of the defect recovery of the cartilages at 4 and 12 weeks after the operation. Values reported as means ± SD for *n* = 6 (* *p* < 0.05, ** *p* < 0.005, *** *p* < 0.001).

The results of the immunohistochemistry analysis are shown in Figure 8A,B. Compared with the 4-week groups, collagen types I and II were evident in the 12-week groups. Cell-seeded hydrogels stained more intensely for both collagens than for empty defect groups.

Type II collagen was primarily distributed in the superficial zone, while type I collagen, presented in all groups, was mainly distributed near the deep and calcified zones. This indicated that the regenerated cartilages were mixtures of hyaline cartilages and fibrocartilages. In addition, there was no significant difference between the cell-seeded hydrogel groups.

To assess cartilage regeneration, we used the histological scoring system described by Wakitani et al.,^47^ with the results presented in Figure 8C. The histological scores were as follows: Empty defect (4.75 ± 2.63), TNC + hADMSCs (7.5 ± 1.64), and TNC + hADMSCs + Etanercept (8.67 ± 2.42) in the 4-week groups. In the 12-week groups, the scores were as follows: Empty defect (9.00 ± 1.826), TNC + hADMSCs (11.00 ± 1.90), and TNC + hADMSCs + Etanercept (10.33 ± 1.37). The standard deviations in all groups were large, so there was mostly no significant difference between each group, except for the TNC + hADMSCs groups between 4 and 12 weeks, and between the Empty defects and TNC + hADMSCs + etanercept groups in 4 weeks.

## 4 Discussion

NC hydrogel was synthesized by graft polymerization of chitosan and NIPAAm monomers (Figure 1A). The radical C-2 amino groups in chitosan chains are suitable targets for grafting with NIPAAm. Polymer grafting reactions modify the physical and mechanical properties to form a hydrogel with thermoresponsive behavior.^38^ The polymerization reaction to synthesize NC in the study was confirmed by the appearance of typical peaks of chitosan and NIPAAm in the FTIR (Figure 1B).^48,49^ NC was further modified using NAC, an altered form of cysteine. Chemical cross-linking between the thiol groups in NAC and the primary amine groups on chitosan resulted in successful thiol modification (TNC), as confirmed by the appearance of a new resonance peak (−CH_2_–SH) in the ^1^H NMR spectrum (Figure 1C).^39^ Chitosan is a widely studied therapeutic polysaccharide. Due to its versatility, it can be modified with various chemical groups, such as thiol groups, and combined with other natural materials to create injectable and thermosensitive formulations for a range of biomedical applications, including drug delivery and tissue engineering.^54−56^ The formation of TNC, a thermosensitive, biocompatible material with enhanced mechanical properties, expands its potential applications as scaffolds for tissue regeneration.^25,39^

From the results of the DSC measurement (Figure 2A), the sol–gel phase transition temperature, known as the lower critical solution temperature (LCST), was about 30–31 °C, similar to the LCST of pNIPAAm. This indicated that the thermosensitive property still existed after synthesis. In addition, the human body temperature is around 37 °C. Clinically, we can prepare the sol-phase hydrogels at room temperature and inject them into the cartilage defect for in situ gelation due to the higher body temperature than the LCST of TNC hydrogels.

SEM observed the interior microstructures of hydrogels. High porosity and interconnected networks are essential for cell nutrition, proliferation, and migration.^57^ As shown in Figure 2B, the porous interior microstructures, interconnected networks, and homogeneous pore distributions were exhibited in hydrogels. The pore size of hydrogels was decreased from about 40 to 10 μm with thiol modification. This result corresponded to the chemical structure with intensive cross-linking after disulfide covalent bond cross-linking. As shown in the SEM micrographs, the thiol-modified hydrogels exhibited a reduction in pore size to approximately 10 μm. Although this pore size may appear small for cell ingrowth and infiltration, it actually helps maintain the structural integrity of the scaffold. Cell ingrowth and infiltration are still possible, particularly for MSCs since their size typically range from 8 to 20 μm.^58^ A 10 μm pore size can provide sufficient space for cells to pass through, infiltrate, attach, and proliferate. Additionally, as the material is a biodegradable scaffold, it gradually degrades over time and allows for gradual infiltration while preserving the tissue architecture.

The mechanical properties of hydrogels plays an essential role in cartilage tissue engineering because of the dynamic loads in the knee joints from body weight. In previous studies, the matrix stiffness in the superficial, deep, and calcified zones of native articular cartilage is 80 kPa, 2.1, and 320 MPa, respectively.^59^ Rheology is an appropriate method to characterize the mechanical properties of hydrogels, since it is sensitive and small sample sizes are needed.^60^ Frequency sweep in rheology is usually performed to evaluate the stability and strength of hydrogels.^61,62^ In this study, mechanical property tests were performed using a hybrid rheometer, as shown in Figure 2C. The storage modulus (*G*′) of the NC and TNC groups was both much higher than the loss modulus (*G*″) at 37 °C, which indicated the gel phase of hydrogels. In addition, the storage modulus (*G*′) of TNC was about 10 times higher than NC, according to the fact that higher *G*′ corresponded to more robust hydrogels;^63^ this result indicated that the mechanical properties of TNC hydrogels were much higher than that of NC hydrogels, which also confirmed that thiol-modified hydrogels successfully improved the mechanical properties by disulfide covalent bond cross-linking. On the other hand, in NC and TNC groups, both storage modulus (*G*′) and loss modulus (*G*″) were significantly dropped down at 25 °C. This indicates that the mechanical properties significantly differed between the gel-like phase at 37 °C and the sol-like phase at 25 °C. Overall, the thiol modification greatly improved the mechanical properties of the hydrogel, following a similar trend to that observed in the study performed by Wu et al., which demonstrated that the mechanical properties of TNC can be enhanced by adjusting the degree of thiol substitution.^25^

Subsequent applications of the hydrogels in this research required confirmation of the cell viability of hydrogels using the CCK8 kit, as shown in Figure 3. The result showed that there was no significant difference between the hydrogel groups and the control group within the same time point, which indicated both hydrogels were considered noncytotoxic according to ISO 10993-5. Moreover, for ideal articular cartilage repair in tissue engineering, the cell source should lower donor site morbidity, quickly expand in large numbers, and have excellent chondrogenic potential.^64,65^ In this study, we used hADMSCs as the cell source due to their chondrogenic potential.

In the present study, evaluations of osteochondral regeneration were usually performed in normal animals. However, traumatic defects and degenerative lesions of articular cartilage may eventually result in osteoarthritis (OA) if patients are in delay of treatments.^1^ Physical diseases such as OA commonly cause osteochondral defects.^2^ The relationship between cartilage defects and osteoarthritis is not independent. Therefore, we evaluated the effects of TNC hydrogels containing hADMSCs on cartilage regeneration in the OA rabbit model.

The chemically induced OA model is easy to induce, repeatable, and suitable for short-term studies and avoids animal infection by eliminating surgery.^5^ In this study, we used the MIA-induced rabbit model, the most commonly used chemically induced model.^11^ After intra-articular injection of MIA solution, the number of chondrocytes decreases, and the histological and morphological articular alterations are similar to human OA changes.^12^ The dosage of MIA solution administered was 4 mg/250 μL in each knee, consistent with prior studies.^41,42^ To evaluate the severity of OA, macroscopic appearance, histological examinations, and the detection of proinflammatory cytokines were performed in this study.

For macroscopic appearance, the irregular and dull surface of the MIA-induced knee, where lesions affecting the entire articular surface, was easy to observe. However, typical OA pathological changes, including hyperemia, swelling in the joint capsule and synovial tissue, and joint effusion,^66^ were not observed in this study. In addition, some rabbits with smooth knee surfaces unsuccessfully induced OA. We could not observe whether OA was successfully induced before seeing the surface of the knee. Previous reports showed that rabbit cartilages exhibit spontaneous healing, particularly in young animals (up to 20 weeks old).^10^ This may be one reason why induced OA lesions were not so severe in the rabbits used in the experiments.

For histological examinations (Figure 4), in the OA group, the irregular surface with fissures and erosions, empty lacunae with hypocellularity, and difficulty in observing superficial and middle zones as well as decreased glycosaminoglycans (GAGs) in the extracellular matrix were shown. These results are in accordance with the effects of MIA, which is a metabolic inhibitor that breaks down the cellular aerobic glycolysis pathway and induces cell death through inhibition of glyceraldehyde-3-phosphate dehydrogenase activity in chondrocytes, as reported in previous studies.^12^ Another study showed that chondrocyte density was decreased, hypertrophic, and disorganized, and clusters were formed to adjust to the changing microenvironments in OA.^67^ However, in the OARSI system, the score of the structure in OA groups was similar to that of the normal group, indicating that the destruction of the articular surface was not evident in this study. In a previous study, researchers reported that MIA-induced arthritis progressed in a manner that was dose- and time-dependent.^68,69^ In future studies, the dose and frequency of MIA administration should be investigated.

To detect the inflammatory cytokines, TNF-α, IL-1β, and IL-6 ELISA kits were used (Figure 4D). In previous studies, it has been proven that TNF-α, IL-1β, and IL-6 are important mediators involved in the pathogenic process of OA, as well as regulators for inflammatory response, and they also play an essential role in the development of synovitis and destruction of the cartilage matrix.^70^ In this study, the levels of TNF-α, IL-1β, and IL-6 in joint fluids of the OA group were significantly increased, indicating that the OA models were successfully induced.

After verification of the OA model, the effects of TNC hydrogels containing hADMSCs with or without etanercept were evaluated in the OA rabbit model. TNF-α is an essential catabolic factor in inflammation and tissue repair for cartilage, which inhibits the ability of mesenchymal stem cells (MSCs) to differentiate into chondroblasts^29^ and leads to the inflammatory and immune response by releasing several cytokines and apoptotic pathway initiation.^31^ Etanercept is one of the most commonly used TNF-α inhibitors. In previous studies, etanercept was used to promote the repair of osteochondral defects in rabbits by blocking TNF-α activity.^36^ In another study, incorporating etanercept into BMSCs enhanced chondrogenesis within an inflammatory microenvironment and effectively counteracted the negative effects of TNF-α in vitro*.*^71^ Therefore, in this study, we used etanercept in OA models to evaluate the enhanced ability of cartilage regeneration.

Macroscopic evaluations, micro-CT analysis, and histological analysis were performed to evaluate osteochondral defect regeneration. For macroscopic evaluations, as shown in Figure 5, based on coverage, color, smooth level, and interaction with the surrounding tissue of neo-tissue, the results showed that 12-week groups were better than 4-week groups, and there was a significant difference between empty defect groups and cell-seeded hydrogels groups. Moreover, the cell-seeded hydrogel with etanercept was better than that without etanercept, but only in the postoperative 4-week groups, which might mean that the effect of the etanercept was only effective in the early stage of cartilage regeneration due to only one subcutaneous injection after the surgery. At the same time, there was no difference between the cell-seeded hydrogel groups in the postoperative 12-week groups. Therefore, in future studies, the dose and frequency of etanercept administration should be investigated. On the other hand, the cartilage regeneration of cell-seeded hydrogels was improved. However, all groups had slightly irregular surfaces of neocartilage, which were caused by OA induction.

Micro-CT was performed to evaluate the qualitative and quantitative bone regeneration levels, as shown in (Figure 6). The values of BV/TV showed similar results to the gross appearance, which is better in the cell-seeded hydrogel groups and the 12-week groups. However, the calculated BV/TV and Tb.Th in the cell-seeded groups were still lower than those in normal rabbits. This result was similar to another research,^72^ which described that the subchondral bone and cartilage formation in the defect area of the OA group was less than that of the non-OA group. Moreover, in a previous study, NAC was used as an osteogenesis promoter to accelerate bone regeneration by activating the differentiation of osteogenic lineages.^73^ This helps explain why the hydrogel groups performed better.

For histological and immunohistochemical analysis (Figures 7 and 8), based on the cell morphology, matrix staining, surface regularity, the thickness of cartilage, regenerated subchondral bone, and integration with adjacent cartilage in neo-tissue, the results were consistent. The cell-seeded hydrogel groups and 12-week groups showed good regeneration. The cell-seeded hydrogel with etanercept was better than without, but only in the postoperative 4-week groups. The reason for this might be because etanercept was subcutaneously injected only once in the present study. A clinical trial evaluating the pharmacokinetics of etanercept found that after a single subcutaneous injection of 25 mg of etanercept, the drug was absorbed slowly, reaching a maximum concentration of 1.37 ± 0.72 μg/mL in the bloodstream at 47 ± 15 h, with a half-life of 80 ± 25 h.^74^ Based on these results, it can be inferred that the half-life of a 125 mg dose would likely remain the same. The half-life of etanercept also explains the lack of significant differences in the gross appearance and micro-CT results of the etanercept group between the 4- and 12-week time points. The effect of incorporating etanercept into the experimental group was only noticeable at the 4-week time point because of the single administration at the start. Therefore, future studies could investigate the effects of multiple administrations of etanercept. Additionally, the standard deviation in all groups was large; therefore, there was mostly no significant difference between each group. This result was caused by the severity of OA differing in each knee, so scoring each section in the same baseline was challenging. The presence of type II and type I collagen in all groups implied that the regenerated cartilages were mixtures of hyaline cartilages and fibrocartilages.

## 5 Conclusions

This study successfully synthesized injectable thermosensitive TNC hydrogels with suitable LCST, porous interior microstructures, and enhanced mechanical properties by disulfide covalent bond cross-linking. To evaluate the effect of TNC hydrogels containing hADMSCs, with or without etanercept, on cartilage regeneration in the OA rabbit model, we established MIA-induced OA models in rabbit knees with verification of macroscopic evaluations, micro-CT analysis, and histological and immunohistochemical assessments. The results demonstrated enhanced cartilage regeneration in the cell-seeded hydrogel, and the addition of etanercept significantly promoted osteochondral repair in the first 4 weeks. Future studies should focus on optimizing the dosage and frequency of etanercept administration. Given these findings, TNC hydrogels combined with chondrogenic stem cells and etanercept show great promise for cartilage tissue engineering in OA models.
